# Validation of the Indonesian resilience evaluation scale in an undergraduate student population

**DOI:** 10.1186/s12889-022-14769-3

**Published:** 2022-12-22

**Authors:** Indira Primasari, Chris M. Hoeboer, Anne Bakker, Miranda Olff

**Affiliations:** 1 Amsterdam UMC location University of Amsterdam, Psychiatry, Meibergdreef 9, Amsterdam, the Netherlands; 2 Amsterdam Public Health, Mental Health, Amsterdam, The Netherlands; 3 Faculty of Psychology, Universitas Indonesia, Depok, Indonesia; 4 ARQ National Psychotrauma Centre, Diemen, The Netherlands

**Keywords:** Assessment, Validation study, Resilience, Undergraduate students

## Abstract

**Background:**

Psychological resilience is an important factor in coping with Potentially Traumatic Events (PTEs) and might mitigate the development of trauma-related disorders. Due to the high risk of natural disasters, criminal activity, and transportation accidents among the Indonesian population, it is critical to assess psychological resilience as a protective factor. This study aimed to validate the Resilience Evaluation Scale (RES) in Indonesian undergraduate students.

**Methods:**

We recruited 327 students (78% female, the mean age is 19.61 (*SD* = 1.24)) between March and June 2020 using convenience sampling, 256 (78.28%) of whom completed the RES twice with an interval of 2 weeks for test-retest reliability purposes. Parallel Analysis and Exploratory Factor Analysis were performed to examine the construct validity of the RES. The internal consistency and the test-retest reliability were assessed using Cronbach Alpha, Pearson Correlations, and Interclass Correlation Coefficients (ICC). Convergent and divergent validity were examined using Pearson Correlations.

**Results:**

EFA analysis yielded a two-factor structure for the final eight-item Indonesian version of RES, which reflected two underlying constructs of resilience: self-confidence and self-efficacy. The Indonesian version of RES demonstrated good internal consistency (α = 0.74–0.82) and test-retest reliability (r = 0.68–0.78; ICC = 0.67–0.78). The result showed that the RES total and subscale scores positively correlated with all criterion variables (resilience, self-efficacy, self-esteem, level of global functioning, and adaptive coping strategy; *r* = 0.27–0.73). RES total and subscale scores negatively correlated with opposite constructs (PTSD, depression, social/work impairment, and maladaptive coping strategy; *r* = - 0.27– -0.46).

**Conclusions:**

The current study showed that the Indonesian RES is a valid and reliable measurement of psychological resilience in Indonesian undergraduate students. The final 8-item Indonesian RES, a freely available resilience instrument, is recommended for future studies and public mental health initiatives in the Indonesian population.

## Introduction

Trauma-related disorders such as depression and post-traumatic stress disorder (PTSD) are prevalent after the experience of a potentially traumatic event (PTE) [1]. However, most people do not develop trauma-related disorders after experiencing a PTE [2]. Social support, optimism, adaptive coping, secure attachment, and resilience are factors that consistently relate to positive post-traumatic outcomes [3]. Particularly, psychological resilience may aid individuals in dealing with PTEs [4] and buffer the impact of PTEs on the development of trauma-related disorders such as PTSD [5, 6], depression, and anxiety [7].

Measuring psychological resilience as a predictive factor might notify the field of intervention andprevention, and public health initiatives [8] as well as research and clinical practice about of the development of trauma-related disorders after a PTE [9]. However, some concerns about the concept of (psychological) resilience have been raised, such as a lack of agreement on the definition and the significant diversity in its operationalization [9, 10]. These problems created substantial obstacles to include (psychological) resilience in intervention and prevention studies [11, 12]. Thus, it is critical to identify resilience’s distinct factors and measure those factors in a reliable and valid way [13].

The Resilience Evaluation Scale (RES) [13] is a free and brief nine-item psychological resilience instrument developed to operationalize psychological resilience based on the Lazarus and Folkman theory of the Cognitive Appraisal Model of Stressful Events [14]. Previous studies indicated that the RES consists of two distinct constructs: (a) self-confidence and (b) self-efficacy, which are valid and reliable for Dutch and English-speaking populations [13]. To our knowledge, there is a limited number of published papers about the validation of RES in other countries, including Indonesia. Furthermore, RES is a novel instrument, and some of the instrument’s psychometric properties have not been tested yet. For example, the test-retest reliability has not been investigated in previous studies, which is essential to prove the stability of the measure [11]. In addition, something that is often overlooked in the validation process is how the measure is culturally acceptable and applicable [11]. Remarkably, little attention has been devoted to validating psychological resilience measurements in non-western cultures [15]. This issue is problematic since psychological resilience might be influenced by the culture and setting where the population is located [16]. Given that previous studies showed that culture might interact with and influence psychological resilience [17], it is crucial to validate the RES in other countries and populations following a cross-cultural validation approach.

In Indonesia, several studies have been carried out to investigate psychological resilience measurements, including the Resilience Scale (RS), the Psychological resilience Quotient (RQ), and the Resiliency Attitude Scale (RAS). However, most of these studies have failed to report standardized cross-cultural adaptation processes and robust psychometric properties. The adaptation process of the RS [18] and the RQ [19] only involved forward translation and expert judgment, but missed backward translation, cognitive interviewing, and further psychometric evaluation. Studies about the RS and RQ have failed to test the construct and convergent-divergent validity. To our knowledge, there is no scientific report about the adaptation and validation study of the RAS in the Indonesian population. Moreover, most of the resilience instruments in Indonesia are not freely available, limiting their accessibility and use in diverse settings.

The present paper aims to validate the RES in Indonesian undergraduate students. The factor structure of the RES in these students was expected to consist of two underlying constructs: self-confidence and self-efficacy. The internal consistency and test-retest reliability of the Indonesian RES were expected to be good (internal consistency: ≥ 0.80, test-retest correlation: ≥ 0.70, and agreement: ≥ 0.75). We also expected to find satisfactory convergent and divergent validity of the Indonesian RES as indicated by a moderately large positive correlation with theoretically related constructs (psychological resilience, self-efficacy, self-esteem, level of global functioning, and adaptive coping strategy) and a moderately large negative correlation with theoretically opposite constructs (PTSD, depression, social-work impairment, and maladaptive coping strategy).

## Methods

### Participants

In total, 327 respondents completed the first survey, and 256 of them completed the RES twice for test-retest reliability purposes between March and June 2020. The mean age of the study participants was 19.61 (*SD =* 1.24). Most of them were female (*n* = 255, 78%), unmarried (*n* = 326, 99.7%), unemployed (*n* = 295, 90.2%), and they attended the Faculty of Health and Medical Sciences (*n* = 254, 77.7%). More detailed characteristics of the participants are presented in Table 1.

**Table 1:** Respondent Characteristics and RES Score

Respondent Characteristics (*N* = 327)		RES Score, mean (SD)
Female, n (%)	255 (78)	22.27 (5.15)
Male, n (%)	72 (22)	23.11 (5.10)
Age, mean (SD)	19.61 (1.24)	
Faculty, *n* (%)		
Social sciences and Humanities	19 (5.81)	21.11 (5.73)
Health and Medical Sciences	254 (77.67)	22.70 (5.02)
Natural, Applied, and Engineering Sciences	54 (16.51)	21.80 (5.48)
Employment, *n* (%)		
Unemployed	295 (90.21)	22.20 (5.00)
Full-time	29 (8.86)	24.76 (5.76)
Part-time	3 (0.91)	25.67 (8.14)
Marital status, *n* (%)		
Married	1 (0.30)	25.00 (−)
Unmarried	326 (99.69)	22.45 (5.15)
Ethnicity, *n*(%)		
Javanese	106 (32.41)	22.25 (5.24)
Sundanese	60 (18.34)	21.38 (5.49)
Balinese	90 (27.52)	22.98 (5.32)
Chinese	24 (7.33)	23.75 (3.77)
Others	47 (14.37)	22.62 (4.61)

### Procedure

The standard cross-cultural adaptation process, according to World Health Organization (WHO) guidelines, was applied to translate the RES from English to Indonesian [20]. A native Indonesian clinical psychologist who is proficient in English conducted the forward translation in the first step. Subsequently, a bilingual (English and Indonesian) expert panel of eight Indonesian psychiatrists and clinical psychologists compared the concepts and language expression in the forward translations and original questionnaire, and then revised the translation. In the next step, a backward translation was performed by a native English professional translator who is highly proficient and knowledgeable in the Indonesian language and culture. The back-translation result was reviewed by the RES developer (CM) [13], the researchers (IP, MO, and AB), and a professional translator before a consensus was achieved for the pre-testing draft.

The next steps of the adaptation process were pre-testing and cognitive interviewing. This procedure aimed to understand the mental process experienced by pre-testing respondents while answering the questionnaire to identify any overt and covert problems in the adaptation process [20]. The pre-testing draft and cognitive interview were piloted with 11 Indonesian participants (18–40 years) representing various gender, education, and professional backgrounds. The final Indonesian RES and adaptation report were submitted to the RES developer (CM) and resulted in the final version of the Indonesian RES.

An online cross-sectional design was conducted among Indonesian undergraduate students. Participants were recruited from public and private universities in Bandung, Surabaya, and Bali in Indonesia using online and offline advertisements. The inclusion criteria for this study were [1] active enrolment as an undergraduate student, [2] age 18 or above, and [3] proficiency in the Indonesian language. Participants who did not sign an informed consent were excluded from the study. The sample size was set to a minimum of 300 respondents based on sample size recommendations for performing Exploratory Factor Analysis (EFA) [21, 22]. Eligible participants received a personal link via email to enter the online questionnaire. Before entering the questionnaire, participants received an information letter with detailed information about the content of the study. Participants were offered to participate in a follow-up study for test-retest reliability purposes. 1 week after the initial study completion, participants who had agreed to participate in the follow-up study received an invitation with a personal link to the second questionnaire set. The personal weblink was closed 1 week later to ensure the follow-up study was answered in the intended time frame. Castor Electronic Data Capture (Castor EDC), a secure and certified online data management platform, was used to collect the data. As an appreciation, participants received a shopping voucher with a value of 25,000 IDR (equal to €1.50). The Health Research Committee, National Institute of Health Research and Development (HREC-NIHRD), and the Ministry of Health Republic of Indonesia approved this study (LB.02.01/2/KE/042/2020).

### Measures

#### Psychological resilience

The RES is a 9-item self-report questionnaire assessing psychological resilience. Respondents rated to what extent each statement applies to them when responding to difficult situations on a 5-point Likert scale. The items are scored from 0 = “completely disagree” to 4 = “completely agree”. The total score ranges from 0 to 36, with higher scores indicating higher psychological resilience. Prior studies indicated good internal consistency (α = 0.78–0.89) and convergent validity of the RES (r = 0.47–0.74; *p* < 0.001) for both total and subscale scores. Meanwhile, the Comparative Fit Index (CFI), Tucker-Lewis Index (TLI), and Root Mean Square Error of Approximation (RMSEA) value using EFA indicating a good model fit (CFI = 0.97–0.98, TLI = 0.97–0.98, RMSEA = 0.06–0.08) [13].

#### Measures for convergent validity

##### Psychological resilience

The Connor-Davidson Resilience Scale (CD-RISC 25) [23] was administrated to measure resilience after experiencing stressful events, traumatic events, or tragedy, including the capacity to adapt to unpleasant situations, to cope with stress and manage unpleasant feelings, and not get disheartened when experiencing disappointment. The CD-RISC 25 contains 25 items on a 5-point Likert scale ranging from 0 = “not true at all” to 4 = “true nearly all of the time.” The total scores range from 0 to 100, with higher scores indicating higher resilience. Previous studies demonstrated a Cronbach‘s alpha of 0.86 to 0.91 for CD-RISC, which indicated good reliability [24, 25].

##### Coping strategy – adaptive coping

The 28-item Brief Coping Orientation to Problem Experienced questionnaire (BRIEF-COPE) [26] – adaptive coping subdomain was used to assess adaptive ways to manage an upsetting life experience. The scale yields scores for adaptive coping (active coping, planning, use of emotional support, use of instrumental support, positive reframing, acceptance, religion, and humor). The items are scored from 1 = “I have not been doing this at all” to 4 = “I have been doing those a lot.” The adaptive coping subdomain score range from 1 to 64. Higher scores of the subdomain reflect higher adaptive coping. Prior studies demonstrated good internal consistency and test-retest reliability of the BRIEF-COPE (α = 0.82–0.91) [27, 28].

##### Self-efficacy

The General Self-Efficacy Scale (GSE) [29] measured perceived self-efficacy regarding adapting and adjusting capacities in both daily exercises and stressful events. The Indonesian version of GSE contains ten items on a 5-point Likert scale [30], ranging from 1 = “not at all true” to 5 = “exactly true.” The full total score ranges from 1 to 50, with higher scores reflecting higher general self-efficacy. Confirmatory Factor Analysis in the previous study showed chi-square = 34.87, df = 26, *p*-value = 0.11444, RMSEA = 0.024, and α = 0.86, which indicated good model fit and reliability [30].

##### Self-esteem

The Rosenberg Self-Esteem Scale (RSE) [31] was used to measure the subjective sense of individual worth or value. The ten items of RSE are rated on a 4-point Likert scale, scored from 0 = “strongly agree” to 3 = “strongly, “disagree,” which items 3, 5, 8, 9, and 10 are reverse scored. Higher total scores of RSE reflect higher self-esteem. Psychometric testing in a previous study showed that the internal consistency of the RSE was good (α = 0.84) [32].

We also included one item from the World Health Organization Quality of Life (WHOQOL-BREF) [33] in measuring participants’ perception of general functioning in their daily life. The item is rated on a 5-point Likert scale, scored from 1 = “very poor” to 5 = “very good.”

#### Measures for divergent validity

##### PTSD symptoms

We used the 20-item self-report PTSD Checklist for DSM-5 (PCL-5) [34] to measure the presence of PTSD symptoms in the past month which corresponds with DSM-5 criteria for PTSD. The items are scored from 0 = “not at all” to 4 = “extremely”. The full total scores extend from 0 to 80, with higher scores reflecting more severe PTSD symptoms. Psychometric testing in a previous study showed that the internal consistency of the PCL-5 was excellent (α = 0.93) [35].

##### Depression symptoms

The Patient Health Questionnaire (PHQ-9) [36], was used to assess depression symptom severity. The Indonesian version of PHQ-9 contains nine items with a 4-point Likert scale, ranging from 0= “not at all” to 3 = “nearly every day,” with total scores ranging from 0 to 27. Higher scores of PHQ-9 reflect a higher severity of depression. The internal consistency and test-retest reliability of the PHQ-9 in previous studies were good (α = 0.72 and ICC = 0.71) [37].

##### Coping strategy – maladaptive coping

The 28-item Brief Coping Orientation to Problem Experienced questionnaire (BRIEF-COPE) [26] – maladaptive coping subdomain was used to assess maladaptive ways to manage an upsetting life experience. The scale yields scores for maladaptive coping (venting, denial, substance abuse, behavioral disengagement, self-distraction, and self-blame) [38]. The items are scored from 1 = “I have not been doing this at all” to 4 = “I have been doing those a lot.” The maladaptive subdomain score ranges from 1 to 48. Higher scores of the subdomains reflect higher maladaptive coping. Psychometric testing in prior studies demonstrated good internal consistency and test-retest reliability of the BRIEF-COPE (α = 0.82–0.91) [27, 28].

##### Social/work impairment

We derived two items (items 24 and 25) from the Clinical-Administered PTSD Scale for DSM-5 (CAPS-5) [39]– Criterion G relating to social and work impairment and presented it to participants as self-report questions to assess the impact of traumatic events on social and work functioning. The two items scored from 0 = mild impact, minimal impairment in social/work functioning“ to 4 = “extreme impact, little or no social/work functioning.” The full total scores extend from 0 to 8, with higher scores reflecting more impairment in social-work functioning.

### Statistical analyses

Data analysis for this study was performed using IBM SPSS Statistics for Windows Version 26.0, R version 3.6.1. and Mplus Version 7.0. Little’s Missing Completely at Random (Little MCAR) Test was conducted to evaluate the indication of missing data type in primary and follow-up studies before running the main analysis. The RES (0.3%), CD-RISC (1.2%), RSE (2.4%), BRIEF-COPE (5.8%), PHQ (4.3%), PCL-5 (4.3%), two items of CAPS-5 (4.6%), and GSE (0.9%) all included some missing data. Missing data for the primary study was successfully imputed using the R package Missforest (Normalized Root mean squared error = 0.37) [40]. Casewise deletion was executed for two respondents with missing data in the follow-up study. Normality and outlier assessment was conducted for assumption and bias checking.

The construct validity of the Indonesian RES was examined using EFA. To determine the number of components of the RES, a parallel analysis (PA) with principal axis factoring was conducted in SPSS version 26 using rawpar.sps syntax [41]. Subsequently, a Maximum Likelihood (ML) extraction was performed, followed by Oblique Promax Rotation, specifying the number of components derived from PA. This is related to the assumption that RES theoretically has two factors likely to be correlated. An EFA with goemin rotation was also performed to assess the goodness of fit of the factor structure (CFI, TLI, and RMSEA) [42].

Internal consistency and the test-retest reliability of the RES total scores and derived factors from the EFA were evaluated. Cronbach’s Alpha, inter-item correlation, item-total correlation, and Cronbach Alpha if the item deleted for subscale scores (based on the EFA result) and total scores were examined to evaluate internal consistency. Pearson Product Moment correlations and Intra-class Correlation Coefficient (ICC) were conducted to measure test-retest correlation and agreement of the RES total scores to assess the measurement stability across time.

The convergent validity of Indonesian RES was assessed by correlating the RES total and subscale (derived from EFA result) scores with the total score of criterion variables resilience, self-efficacy, self-esteem, adaptive coping strategy, and global functioning. Furthermore, the divergent validity of the Indonesian RES was assessed by correlating the RES total score and subscale (derived from EFA result) scores with PTSD, depression, social/work impairment, and maladaptive coping. A Pearson product-moment correlation was used to analyze both convergent and divergent validity.

## Results

### Construct validity

The initial PA indicated a three-factor solution as three factors contained eigenvalues above the 95th percentile. However, based on the scree plot examination, the slope of the generated scree plot drastically shifted between the second and third factor, with the eigenvalue of the third factor of only 0.24 and only slightly above the 95th percentile (0.17), which indicated that a two-factor solution might be better. Subsequently, we explored the two-factor and three-factor solutions as indicated by the PA. For the three-factor solution, we found that item 8 (*I can handle a lot at the same time*) showed a low factor loading on the third factor (λ = 3.82), and the Cronbach’s Alpha total score was improved (α = 0.813 to α = 0.823) if item 8 was deleted. We also found that the third factor consisted of only two items (item 6 and item 8) which theoretically might lead to an identification problem. For the two-factor solution, we found that item 8 did not load on any of the two factors, indicating that the item was insufficient to have a relational construct with either factor. Therefore, item 8 was dropped from the subsequent analysis. The PA was re-run without item 8, yielding a two-factor solution with eigenvalues 2.97 and 0.43 above the 95th percentile. The EFA showed a two-factor solution with eigenvalues 3.59 and 1.04, respectively (eigenvalues for the third to eighth factor were lower than one, ranging from 0.33–0.74). All factor loadings on the two factors were significant, with sufficient total variance explained (57,96%), and no cross-loadings were observed. The CFI, TLI, and RMSEA indicated a good model fit (CFI = 0.98, TLI = 0.96, and RMSEA = 0.05). This model, without item 8, was selected as the best factor solution for the Indonesian version of RES. Factor 1 (items 1, 7, and 9) was named “self-confidence,” and Factor 2 (items 2, 3, 4, 5, and 6) was named “self-efficacy” (Table 2).

**Table 2:** Factor loadings for the two-factor solution model of Indonesian RES with eight items

	RES ITEM	Factor 1 (self-confidence)	Factor 2 (self-efficacy)
1	*Saya memiliki rasa percaya diri* (I have confidence in myself)	0.665	
2	*Saya mampu menyesuaikan diri dengan mudah dalam situasi sulit* (I can easily adjust in a difficult situation)		0.462
3	*Saya mampu bertahan dengan gigih dalam situasi sulit* (I am able to persevere)		0.774
4	*Setelah mengalami hambatan, saya dapat dengan mudah bangkit kembali* (After setbacks, I can easily pick up where I left off)		0.386
5	*Saya tahan banting (tangguh)* (I am resilient)		0.658
6	*Saya mampu mengatasi dengan baik berbagai masalah yang muncul secara tidak terduga* (I can cope well with unexpected problems)		0.407
7	*Saya menghargai diri saya sendiri* (I appreciate myself)	0.717	
9	*Saya percaya pada diri saya sendiri* (I believe in myself)	0.877	

### Reliability

Cronbach’s alpha coefficients for RES total score and subscale scores (Self-Confidence and Self-Efficacy score) were good and did not improve if an item was deleted. Test-retest correlations of RES total score and RES Self-Confidence between the primary and follow-up studies indicated high test-retest reliability. In addition, the test-rest correlation of RES Self-Efficacy between the primary and follow-up studies indicated moderate test-retest reliability. The ICC analysis of RES total score and RES Self-Confidence subscale score indicated excellent absolute agreement between the primary and the follow-up study with its 95% confidence interval ranging between 0.710–0.815 (total score) and 0.725–0.823 (self-confidence subscale), indicating good to excellent test-retest reliability. The ICC analysis on the RES Self-Efficacy subscale score showed good absolute agreement between primary and follow-up studies. The 95% confidence interval ranged between 0.598–0.735, indicating good test-retest reliability (Table 3).

**Table 3:** Internal Consistency & Test-Retest Reliability (Total score and Subscale scores)

RES	Cronbach’s α	Range Inter-item Correlation	Range Item-Total Correlation	Test-Retest Correlation	Absolute Agreement (ICC – Lower-Upper Bound)
RES Total Score (8 items)	0.823	0.248–0.619	0.488–0.668	0.761^a^	0.768 (0.710–0.815)
RES Self-Confidence Subscale (Item 1, 7, 9)	0.798	0.488–0.619	0.607–0.708	0.781^a^	0.779 (0.725–0.823)
RES Self-Efficacy Subscale (Item 2, 3, 4, 5, 6)	0.737	0.259–0.488	0.466–0.545	0.679^a^	0.672 (0.598–0.735)

^a^Pearson Correlation coefficient significant at the .01 level

### Convergent and divergent validity

There was a significant positive correlation between the RES total score, subscale scores, and all criterion variables (resilience, self-efficacy, self-esteem, level of global functioning, and adaptive coping strategy). The RES total score showed the highest positive correlation with the CD-RISC 25 (resilience). The RES Self-Confidence Subscale showed the highest positive correlation with the Rosenberg Self-Esteem Scale total score (Self-Esteem). The RES Self-Efficacy Subscale showed the highest positive correlation with the General Self-Efficacy Scale total score (Self-Efficacy). A significant negative correlation was found between the RES total score, subscale scores, and expected different construct variables (PTSD, depression, social/work impairment, and maladaptive coping strategy) (Table 4).

**Table 4:** Convergent & Divergent Validity (Total score and Subscale scores)

	RES Total (8 items)	RES Self-Confidence Subscale (Item 1, 7, 9)	RES Self-Efficacy Subscale (Item 2, 3, 4, 5, 6)
Convergent Validity			
Resilience (CD-RISC 25)	0.739^a^	0.659^a^	0.649^a^
Self-Efficacy (General Self-Efficacy Scale)	0.715^a^	0.572^a^	0.683^a^
Self-Esteem (Rosenberg Self-Esteem Scale)	0.655^a^	0.687^a^	0.489^a^
Adaptive Coping (Brief-COPE – Adaptive Coping Subdomain)	0.318^a^	0.274^a^	0.286^a^
Global Functioning (WHO Quality of Life-BREF)	0.475^a^	0.485^a^	0.365^a^
Divergent Validity			
PTSD (PCL-5)	-0.390^a^	-0.401^a^	- 0.297^a^
Depression (PHQ-9)	-0.410^a^	-0.463^a^	-0.279^a^
Social/Work impairment (CAPS-5 – 2 items)	-0.399^a^	-0.377^a^	-0.333^a^
Maladaptive Coping (Brief-COPE – Maladaptive Coping Subdomain)	-0.413^a^	-0.413^a^	- 0.325^a^

^a^Pearson Correlation coefficient significant at the .01 level

## Discussion

This study aimed to translate the RES into Indonesian and test the psychometric properties in an Indonesian undergraduate student sample. Overall findings in this present work demonstrated good validity and reliability of the instrument.

The original RES proposed a two-factor structure, *self-confidence*, and *self-efficacy*, as the construct of resilience based on the Lazarus and Folkman model [13]. We identified two underlying constructs of resilience for the final 8-item version of Indonesian RES: *self-confidence* and *self-efficacy*. This corresponds to the English and Dutch versions of the RES [13]. The items for the two constructs also matched with the previous study, except for item 8, which did not substantially contribute to any factor. Three items (items 1, 7, 9) clustered on the construct self-confidence, and five items (items 2, 3, 4, 5, and 6) clustered on the construct self-efficacy. Therefore, we recommend measuring resilience using the final 8-item version of the RES in Indonesian undergraduate students. In this version, item 8 (*I can handle a lot at the same time*), which was associated with the construct *self-efficacy* in a previous study [13], was eliminated from the questionnaire. This question might not add something to an Indonesian student population where everyone has to handle a lot simultaneously. Put differently, it might not differentiate students with high self-efficacy from those with low self-efficacy because part of being a student includes taking schoolwork and being with one’s family at the same time. In the Indonesian culture, multitasking is considered a norm that has been continuously expected from each individual [43], particularly for women, who were the majority of respondents in this study. Meanwhile, in the Western population, where the RES was validated so far, multitasking has a more positive connotation associated with self-efficacy. Future studies might check whether this item contributes to measuring resilience in another Indonesian population. In addition, the Indonesian RES also showed high test-retest reliability in this study, proving that the measurement is stable over time [44].

The current study also evaluated the convergent and divergent validity of the Indonesian RES in undergraduate students. The 8-item RES and the subscales (self-confidence and self-efficacy) demonstrated a positive relationship with all measures theoretically assumed to be related (resilience, self-esteem, self-efficacy, adaptive coping, and global functioning). These findings indicate that the Indonesian RES and related/other measurements capture a common construct as expected. Additionally, the results of this study are consistent with prior research. A previous study found that self-esteem is considered a protective factor for resilience, which helps individuals cope with adverse life events more positively and confidently [45]. A previous study also showed that higher resilience is related to higher psychosocial functioning in individuals [46]. Notably, the relationship between the Indonesian RES and adaptive coping was relatively weak (just above the minimum recommended value for convergent validity). A possible explanation is that although adaptive coping strategies such as planning and the search for emotional and instrumental support are more likely to reduce emotional fatigue and improve resilience, their effect can be ineffective in situations where problems are uncontrollable, and conditions cannot be alternated [47].

Furthermore, the total 8-item RES and the subscales (self-confidence and self-efficacy) showed significant negative correlations with all expected opposite constructs (PTSD, depression, social/work impairment, and maladaptive coping strategy). All correlations were negative, indicating that Indonesian RES and other measures are discriminated against each other and refer to distinct constructs. This finding is consistent with previous research that found a negative relationship between resilience and psychopathology (anxiety and depression symptoms) [48].

We note several strengths of this study that should be highlighted. This study included a rigorous cultural adaptation process involving numerous Indonesian mental health experts aligned with WHO guidelines. Conducting a careful cultural adaptation process is essential for a valid and reliable measurement in a novel language [49]. Furthermore, we uncovered novel evidence for the test-retest reliability of the Indonesian RES both on a group and individual level, which is often neglected in validation studies [50].

We also recognize several limitations. Firstly, convenience sampling, online advertisement, and online data collection are limited to respondents with an internet connection, potentially excluding respondents living in most remote areas. Secondly, this study focused on undergraduate students, and the majority of them were women and unmarried, which restricted the generalization to other Indonesian populations.

Considering the cultural and social-economic background of the Indonesian people, future studies can further explore the two-factor structure of the 8-item Indonesian RES and convergent, divergent, and discriminant validity with another sample/ other samples. Future studies are also suggested to explore whether multitasking is a norm for other Indonesian populations with regard to psychological resilience. A longitudinal study that follows individuals after a traumatic event to determine whether psychological resilience accurately predicts post-traumatic outcomes, is also recommended for future studies. This approach could discover how the Indonesian RES could identify significant changes, dynamics, and mechanisms in resilience from time to time, both in individual and group contexts, and the factors that influence these changes [51].

## Conclusion

In summary, this study set out to validate the Indonesian version of the RES, which can be used by Indonesian undergraduate students. A comprehensive cross-cultural adaptation was conducted to ensure that the construct of psychological resilience has been culturally captured. According to the findings of this study, the Indonesian RES is a valid and reliable measure of psychological resilience in Indonesian undergraduate students. Given that Indonesia is one of the world’s most disaster-prone countries, contributing to the higher risk of experiencing PTEs and trauma-related disorders, this study contributes to the larger Indonesian population by providing a resilience instrument for future studies. The final 8-item Indonesian RES is recommended for research and clinical practice, which might be beneficial to further research and public mental health initiatives.

## Data Availability

The datasets generated and/or analyzed during the current study are not publicly available due to ethical restrictions but are available from the corresponding author on reasonable request.

## Abbreviations

PTSD: Post-traumatic Stress Disorder
PTE: Potentially Traumatic Event
RES: Resilience Evaluation Scale
RS: Resilience Scale
RQ: Psychological resilience Quotient
RAS: Resiliency Attitude Scale
EDC: Electronic Data Capture
WHO: World Health Organization
EFA: Exploratory Factor Analysis
CD-RISC 25: Connor-Davidson Resilience Scale
BRIEF-COPE: Brief Coping Orientation to Problem Experienced
GSE: General Self-Efficacy Scale
RSE: Rosenberg Self-Esteem Scale
WHOQOL-BREF: World Health Organization Quality of Life Scale
PCL-5: PTSD Checklist for DSM-5
PHQ-9: Patient Health Questionnaire
CAPS-5: Clinical-Administered PTSD Scale for DSM-5
MCAR: Missing Completely at Random
PA: Parallel Analysis
ML: Maximum Likelihood
CFI: Comparative Fit Index
TLI: Tucker-Lewis Index
RMSEA: Root Mean Square Error of Approximation
ICC: Intra-class Correlation Coefficient

## References

[1] Kessler RC, Aguilar-Gaxiola S, Alonso J, Benjet C, Bromet EJ, Cardoso G, Degenhardt L, de Girolamo G, Dinolova RV, Ferry F, Florescu S (2017). Trauma and PTSD in the WHO world mental health surveys. Eur J Psychotraumatol.

[2] Friedberg A, Malefakis D (2018). Resilience, trauma, and coping. Psychodyn Psychiatry.

[3] Nugent NR, Sumner JA, Amstadter AB (2014). Resilience after trauma: from surviving to thriving. Eur J Psychotraumatol.

[4] Campodonico C, Berry K, Haddock G, Varese F. Protective factors associated with post-traumatic outcomes in individuals with experiences of psychosis. Front Psychiatry. 2021:2164. 10.3389/fpsyt.2021.735870. 10.3389/fpsyt.2021.735870 PMC8666594 34912247

[5] Matheson K, Asokumar A, Anisman H (2020). Resilience: safety in the aftermath of traumatic stressor experiences. Front Behav Neurosci.

[6] Wang S, Shi X, Chen X, Zhu Y, Chen H, Fan F (2021). Earthquake exposure and PTSD symptoms among disaster-exposed adolescents: a moderated mediation model of sleep problems and resilience. Front Psychiatry.

[7] Leys C, Kotsou I, Shankland R, Firmin M, Péneau S, Fossion P (2021). Resilience predicts lower anxiety and depression and greater recovery after a vicarious trauma. Int J Environ Res Public Health.

[8] Magruder KM, McLaughlin KA, Elmore Borbon DL (2017). Trauma is a public health issue. Eur J Psychotraumatol.

[9] Denckla CA, Cicchetti D, Kubzansky LD, Seedat S, Teicher MH, Williams DR, Koenen KC (2020). Psychological resilience: an update on definitions, a critical appraisal, and research recommendations. Eur J Psychotraumatol.

[10] Southwick SM, Bonanno GA, Masten AS, Panter-Brick C, Yehuda R (2014). Resilience definitions, theory, and challenges: interdisciplinary perspectives. Eur J Psychotraumatol.

[11] Windle G, Bennett KM, Noyes J (2011). A methodological review of resilience measurement scales. Health Qual Life Outcomes.

[12] Chmitorz A, Kunzler A, Helmreich I, Tüscher O, Kalisch R, Kubiak T, Wessa M, Lieb K (2018). Intervention studies to foster resilience–a systematic review and proposal for a resilience framework in future intervention studies. Clin Psychol Rev.

[13] Van der Meer CA, Te Brake H, Van der Aa N, Dashtgard P, Bakker A, Olff M (2018). Assessing psychological resilience: development and psychometric properties of the English and Dutch version of the resilience evaluation scale (RES). Front Psychiatry..

[14] Lazarus RS, Folkman S (1984). Stress, appraisal, and coping.

[15] Ungar M (2008). Resilience across cultures. Br J Soc Work.

[16] Masten AS, Lucke CM, Nelson KM, Stallworthy IC (2021). Resilience in development and psychopathology: multisystem perspectives Ann. Annu Rev Clin Psychol.

[17] Raghavan SS, Sandanapitchai P (2019). Cultural predictors of resilience in a multinational sample of trauma survivors. Front Psychol.

[18] Karsiyati (2012). Hubungan Resiliensi dan Keberfungsian Keluarga Pada Remaja Pecandu Narkoba Yang Sedang Menjalani Pemulihan [bachelor's thesis on the Internet].

[19] Syifa MA. Hubungan Resiliensi dengan Stres pada Taruna Tingkat I di Sekolah Tinggi Perikanan [bachelor's thesis on the Internet]. Jakarta (IND): Universitas Negeri Jakarta; 2019 [cited 2022 Nov 16]. Available from: http://repository.unj.ac.id/3099/1/SKRIPSIMUTHIAAMALIASYIFA1125152071.pdf.

[20] World Health Organization (2016). Management of substance abuse: process of translation and adaptation of instruments [internet].

[21] Comrey AL, Lee HB (1992). A first course in factor analysis.

[22] Thompson B (2004). Exploratory and confirmatory factor analysis: understanding concepts and applications.

[23] Connor KM, Davidson JR (2003). Development of a new resilience scale: the Connor-Davidson resilience scale (CD-RISC). Depress Anxiety.

[24] Ratna JMJ (2015). The impact of an antenatal resilience and optimism workshop on postnatal depressive symptoms [dissertation on the internet].

[25] Almasyhur AF (2021). Uji Validitas Instrumen Connor-Davidson resilience scale 25 (CD-RISC 25) Versi Bahasa Indonesia [master's thesis on the Internet].

[26] Carver CS (1997). You want to measure coping, but your protocol is too long: consider the brief cope. Int J Behav Med.

[27] Utami ELT (2018). Strategi Koping Dan Distres Psikologis Pada Mahasiswa Fakultas Kedokteran Universitas Indonesia [bachelor's thesis on the internet].

[28] Amanda R (2014). Hubungan antara Gratitude dengan Coping pada Mahasiswa Penerima Beasiswa Pendidikan bagi Mahasiswa Berprestasi (Bidikmisi) [bachelor's thesis on the internet].

[29] Schwarzer R, Jerusalem M, Weinman J, Wright S, Johnston M (1995). Generalized self-efficacy scale. Measures in health psychology: a user's portfolio. Causal and control beliefs.

[30] Novrianto R, Marettih AKE, Wahyudi H (2019). Validitas Konstruk Instrumen general self efficacy scale Versi Indonesia. J Psikol.

[31] Rosenberg M, Rosenberg M (1965). Self-esteem scale. Appendix D. Society and the Adolescent Self-Image.

[32] Hanani CA (2019). Pengaruh Self-esteem terhadap Resiliensi pada Mahasiswa Tahun Pertama Program Studi Kedokteran. [bachelor's thesis on the internet].

[33] Harper A, Power M, Orley J, Herrman H, Schofield H, Murphy B (1998). Development of the World Health Organization WHOQOL-BREF quality of life assessment. Psychol Med.

[34] Weathers FW, Litz BT, Keane TM, Palmieri PA, Marx BP, Schnurr PP (2013). The PTSD checklist for DSM-5 (PCL-5).

[35] Asti GDA (2015). De Psychometrische Eigenschappen van de Indonesische Versie van de PTSD Checklist for DSM-5 (PCL-5-Ind) in Javaanse Populatie [master's thesis].

[36] Kroenke K, Spitzer RL (2002). The PHQ-9: a new depression diagnostic and severity measure. Psychiatr Ann.

[37] van der Linden A (2019). Cross-cultural validation of the patient health questionnaire (PHQ-9) in Bahasa Indonesia to measure depression among people affected by leprosy in Central Java, Indonesia [master's thesis on the internet].

[38] Meyer B (2001). Coping with severe mental illness: relations of the brief COPE with symptoms, functioning, and well-being. J Psychopathol Behav Assess.

[39] Weathers FW, Bovin MJ, Lee DJ, Sloan DM, Schnurr PP, Kaloupek DG, Keane TM, Marx BP (2018). The clinician-administered PTSD scale for DSM–5 (CAPS-5): development and initial psychometric evaluation in military veterans. Psychol Assess.

[40] Stekhoven DJ, Bühlmann P (2012). MissForest—non-parametric missing value imputation for mixed-type data. Bioinformatics..

[41] O'connor BP (2000). SPSS and SAS programs for determining the number of components using parallel analysis and Velicer's MAP test. Behav Res Methods Instrum Comput.

[42] Muthén LK, Muthén BO. Mplus User’s Guide. Seventh Ed. Muthén & Muthén. Los Angeles.1998

[43] Wijaya LH, Rohimi (2020). Perempuan dan Pemberdayaan Ekonomi Masyarakat (Teori, Entitas, dan Perannya Di dalam Pekerjaan Sektor Informal).

[44] Matheson GJ (2019). We need to talk about reliability: making better use of test-retest studies for study design and interpretation. PeerJ..

[45] Li J, Chen YP, Zhang J, Lv MM, Välimäki M, Li YF, Yang SL, Tao YX, Ye BY, Tan CX, Zhang JP (2020). The mediating role of resilience and self-esteem between life events and coping styles among rural left-behind adolescents in China: a cross-sectional study. Front Psychiatry..

[46] Wambua GN, Kilian S, Ntlantsana V, Chiliza B (2020). The association between resilience and psychosocial functioning in schizophrenia: a systematic review and meta-analysis. Psychiatry Res.

[47] de la Fuente J, Fernández-Cabezas M, Cambil M, Vera MM, González-Torres MC, Artuch-Garde R (2017). Linear relationship between resilience, learning approaches, and coping strategies to predict achievement in undergraduate students. Front Psychol.

[48] da Silva-Sauer L, de la Torre-Luque A, Smith B, Lins M, Andrade S, Fernández-Calvo B (2021). Brief resilience scale (BRS) Portuguese version: validity and metrics for the older adult population. Aging Ment Heal.

[49] Sousa VD, Rojjanasrirat W (2011). Translation, adaptation and validation of instruments or scales for use in cross-cultural health care research: a clear and user-friendly guideline. J Eval Clin Pract.

[50] Berchtold A (2016). Test-retest: agreement or reliability?. Methodological Innov.

[51] Crane MF, Lewis V, Cohn A, Hodson SE, Parslow R, Bryant RA (2012). A protocol for the longitudinal study of psychological resilience in the Australian Defence force. J Mil Veterans Heal.

